# Fructose Consumption During Pregnancy Influences Milk Lipid Composition and Offspring Lipid Profiles in Guinea Pigs

**DOI:** 10.3389/fendo.2020.00550

**Published:** 2020-08-11

**Authors:** Erin Vanessa LaRae Smith, Rebecca Maree Dyson, Mary Judith Berry, Clint Gray

**Affiliations:** ^1^Department of Paediatrics and Child Health, University of Otago, Wellington, New Zealand; ^2^Centre for Translational Physiology, University of Otago, Wellington, New Zealand

**Keywords:** maternal fructose, developmental programming, milk composition, hepatic lipids, free fatty acids

## Abstract

Excess dietary fructose is a major public health concern (1–4). Evidence shows increased fructose intake can cause insulin resistance, hepatic *de novo* lipogenesis, hypertriglyceridemia, obesity and non-alcoholic fatty liver disease (NAFLD) (5–9). However, little is known about the effects of fructose during pregnancy and its influence on offspring development and predisposition to later-life disease. To determine whether moderately increased maternal fructose intake could have health consequences on offspring, we have investigated the effects of 10% w/v fructose water intake during preconception and pregnancy. Female Dunkin Hartley guinea pigs were fed a control diet (CD) or fructose diet (FD;10% kcal from fructose) *ad-libitum* 60 days prior to mating and throughout gestation. Offspring were culled at weaning, day 21 (d21). Compared to CD dams, FD dams had altered glucose metabolism and increased milk free fatty acid content. Matsuda-DeFronzo insulin sensitivity index (M-ISI) from OGTT plasma showed no significant difference in whole-body insulin sensitivity between FD and CD dams 60 days post-dietary intervention and during midgestation. Fetal exposure to increased maternal fructose resulted in offspring with significantly altered serum free fatty acids at days 0, 7, 14, and 21 [including pentadecanoic acid (15:0), dma16:0, margaric acid (17:0) palmitoleic acid, total omega-7 and total saturates], increased levels of uric acid and triglycerides were also observed at d21. We have demonstrated that increased fructose intake during pregnancy can cause significant changes in maternal metabolic function and milk composition, which alters offspring metabolism. Taken together, these changes in pregnancy outcomes and feto-maternal condition may underlie their offspring's predisposition to metabolic dysfunction during later-life.

## Introduction

Excess dietary fructose intake is a major public health concern (1–4). Under normal physiological conditions, when the liver metabolizes fructose, it favors lipogenesis. It has also been shown that when the liver metabolizes fructose excess, it can result in metabolic dysregulation resulting in hyperlipidemia, insulin resistance, obesity, diabetes, cardiovascular disease and non-alcoholic fatty liver disease (5–9). The World Health Organization (WHO) recommends simple sugars from processed foods and sugar-sweetened beverages should be <10% of total daily caloric intake (10). It is currently estimated that 18–25% of total daily calorie intake comes from simple sugars in a Westernized diet (11). However, there is a paucity of data examining the impact of increased fructose intake before and during pregnancy and subsequent adverse effects on lactation, fetal development and offspring metabolic function.

Metabolic adaptations during pregnancy are necessary for appropriate metabolic demands supporting appropriate growth and development of the fetus and preparation for lactation. Human epidemiological studies and animal models have shown that poor quality nutrition during fetal growth can predispose the offspring to long-term health consequences (12, 13). Unbalanced maternal nutrition, in particular, overnutrition, can have permanent effects on her offspring's organ structure and function, predisposing them to adult-onset of non-communicable disease such as; obesity, diabetes, NAFLD and cardiovasclar risk factors (14–17). The contemporary western women consuming high levels of fructose before and/or during pregnancy may alter critical phases of pregnancy, such as embryogenesis, fetal-placental development, and milk production and quality (15, 18).

Previous research in rodents has demonstrated that maternal consumption of 10% fructose in water during pregnancy and lactation resulted in maternal hyperglycemia, hyperinsulinemia and hypertriglyceridemia, which were associated with significantly elevated plasma insulin in offspring at weaning, suggesting offspring susceptibility to diabetes during adulthood (19). Further rodent studies demonstrated that 20% of caloric intake from fructose during gestation resulted in maternal hyperinsulinemia and sex-specific effects in offspring with female offspring having higher plasma leptin and glucose and displaying greater vulnerability to metabolic disturbances in neonatal life than male offspring (20). Additionally, we have previously shown sex-specific cardio-metabolic differences in the offspring of maternal 10% w/v fructose-fed dams (11). Some studies have reported that high fructose intake alters beta-oxidation, increases free fatty acids (FFAs) and triglycerides, causing dyslipidemia, hepatic lipid accumulation and insulin resistance (5, 21, 22). It is essential to determine how the vertical transmission of such deleterious metabolic effects might increase *de novo* lipogenesis, fatty acid acylation and subsequent metabolic programming in offspring. Therefore, the current study aimed to characterize the phenotypic effects of excess maternal fructose consumption on maternal physiology, metabolism, milk quality and determine the impact upon physiology and metabolic status in young offspring.

## Materials and Methods

### Ethics

All studies were conducted with the prior ethical approval of the University of Otago Wellington Animal Ethics Committee (AEC 7-15) and performed in line with the standards described in the Guide for the Care and Use of Laboratory Animals, 8th Edition, and the National Animal Ethics Advisory Commission, New Zealand (23). Results are reported according to the ARRIVE guidelines (24).

### Animal Model

All animals were housed in polypropylene cages (Tecniplast, Australia) lined with untreated pine wood shavings in a sound-insulated room under a 12 h day/night light cycle within a temperature (18–23°C) and humidity controlled facility. Nineteen Virgin Dunkin Hartley females were randomly allocated to two different experimental groups: (1) Control diet (CD; *n* = 10) and (2) Fructose-diet (FD; *n* = 9). All guinea pigs received standard guinea pig chow (guinea pig pellets; Sharpes Stock Feeds, NZ) (Table 1), hay ~10 g, half of fresh silverbeet and ~18 g carrots daily. Millipore filtered water supplemented with 1.2 g/L of ascorbic acid was available *ad libitum*. FD dams water was additionally supplemented with 10 g/ml of D-fructose (Sigma Aldrich, USA) to create 10% fructose water from 12 weeks (w) to term delivery. Dam and offspring weights, water and food intake, as well as caloric intake, were calculated daily. Kcal intake from food was calculated daily using the manufacturer's feed formulation guide (Table 1).

**Table 1:** Guinea pig pellet nutrition facts and ingredients from Nutritech (Nutritech International Ltd., NZ).

**Feedstuff**	**Fresh weight**	**(%)**	**Nutrient**	**Amount**	**DM 100%**
**FORMULATION SUMMARY**					
Broll	381.00	38.10	Bulk	1.00	1.14
Lucerne pellets	300.00	30.00	Dry Matter (%)	88.02	100.00
Barley	220.00	22.00	RUM_MER (MJ/kg)	10.03	11.40
Soya, extr, 47%	55.00	5.50	Crude Protein (%)	15.90	18.06
Molasses, cane	20.00	2.00	Oil A (%)	2.61	2.96
Limestone	20.00	2.00	Crude Fiber (%)	12.37	14.05
Salt	2.00	0.20	ADF (%)	15.31	17.39
Rabbit Premix	2.00	0.20	NDF (%)	29.87	33.93
Rabbit Premix	2.00	0.20	NDF (%)	29.87	33.93
Total	1,000.00 kg	100.00	Starch (%)	20.92	23.77
			Starch + Sugar (%)	26.57	30.19
			Calcium (%)	1.32	1.50
			Phosphorus (%)	0.55	0.62
			Magnesium (%)	0.24	0.27
			Sodium (%)	0.11	0.13

### Mating and Postnatal Care

At 6-months of age, dams were housed in a non-lineage harem with a single boar during estrous for a maximum of 72 h to ensure timed mating. Following mating, dams returned to individual cages and continued their experimental diets throughout gestation. After mating, abdominal ultrasounds were performed by experienced technicians to confirm pregnancy, detectable 3 weeks post-mating. Dams delivered spontaneously, and all litters were maintained at four pups following delivery. Total groups included CD males (*n* = 7); FD males (*n* = 7); CD females (*n* = 7); and FD females (*n* = 7). Excess pups were humanely euthanized by intraperitoneal injection of 0.5 ml of Pentobarbital. Each dam was housed with her pups until weaning (d21). Dams were weighed weekly, signs of lactation tracked daily and all pups weighed daily.

### Dam and Offspring Oral Glucose Tolerance Tests and Blood Glucose Analysis

Oral glucose tolerance tests (OGTT) were performed on dams and offspring. In brief, all animals were fasted for 14 h overnight with *ad libitum* access to water. The animals were wrapping in a towel to limit movement and stress. A baseline blood collection (250 μl) was collected from the auricular vein. A standardized oral dextrose load (1,000 mg/kg) was measured then administered orally using a 5 ml syringe. Serial blood collections for glucose and insulin at 15, 30, 45, 60, 75, 90, 120, and 180 min for dams and 30, 60, 120, and 180 min for the offspring, due to their smaller size. In addition, offspring blood samples (50 μl) were taken for glucose at day 0 (prior to suckling), 7 and 14, following a 4 h fast during which time food, but not water, was withdrawn. All blood samples were collected via the auricular vein, placed on ice, then centrifuged at 4,000 rpm for 15 min. The plasma layer was extracted and stored at −80°C pending analysis. The Matsuda-DeFronzo Insulin Sensitivity Index (M-ISI) was used to evaluate whole-body insulin sensitivity in dams (25, 26). The Matsuda Index

$$\begin{array}{l} 1,000/\surd [(\text{fasting plasma glucose }(\text{mg/dl})\\ \;\times \text{fasting plasma insulin }(\mu \text{U/ml}))\\ \;\times (\text{mean glucose }(\text{mg/dl})\\ \;\times \text{mean insulin }(\mu \text{U/ml})]. \end{array}$$

### Dam Milk Free Fatty Acid Analysis

Dam's milk was extracted within 4 h of the birth of the last pup. Approximately 40 μl of milk was applied to a Dried Milk Spot (DMS) PUFAcoat? card (University of Adelaide, Australia). PUFAcoat? cards were dried at room temperature for 1 h and stored at room temperature until further analysis (27, 28).

### Offspring Serum Free Fatty Acids Analysis

At d0 (prior to feeding), d7, d14, and d21 ~40 μl of blood was collected from the auricular vein and placed on a Dried Blood Spot (DBS) PUFAcoat? card (University of Adelaide, Australia), dried at room temperature for ~1 h and stored at room temperature until further analysis.

### PUFAcoat Milk and Offspring Serum Free Fatty Acid Analysis

Both milk and offspring serum measurement for free fatty acids were performed using a modified direct transesterification method (29). Fatty acids were transmethylated to fatty acid methyl esters (FAME) using 2 ml of 1% (v/v) sulphuric acid H_2_SO_4_ in anhydrous methanol in a 5 ml sealed vial and heated for 3 h at 70°C. This procedure allows for fatty acids to be released from structural lipids into a total fatty acid pool. The sequential FAME was extracted into heptanes for further gas chromatography analysis (27, 28, 30). Sample FAME identification and quantification were achieved by comparing the retention times and peak area values to those of the commercial lipid standards (Nu-Chek-Prep, USA) using the Hewlett-Packard Chemstation data system (27, 28, 30, 31).

### d21 Offspring Tissue Collection

Immediately prior to weaning (d21), offspring were fasted for 14 h. Guinea pigs were euthanised by intracardiac injection of sodium pentobarbital (>100 mg/kg) (32) and brain, plasma, urine, liver, quadracep muscle, visceral and subcutaneous fat depots were collected, weighed, snap-frozen in liquid nitrogen and stored at −80°C or fixed in 10% formalin phosphate buffer for later analysis.

### Plasma Metabolic Analysis

Maternal and offspring plasma were analyzed by Cobas c311 autoanalyzer (Roche) according to manufacturer instructions for cholesterol (CHOL), triglycerides (TAG) low-density lipoprotein cholesterol (LDL), high-density lipoprotein cholesterol (HDL) and uric acid (UA). Maternal and offspring plasma were analyzed for insulin by ELISA (Abclonal, USA) according to the manufacturer's instructions.

### Statistical Analysis

Statistical analyses were performed using IBM SPSS Statistics 25 software (IBM, USA). All data were graphed using Prism 8 software (GraphPad Software Inc., USA). Dam growth, caloric intake, stillbirth/death during delivery to live birth ratio, litter size, Matsuda-DeFronzo Insulin Sensitivity Index (M-ISI) during OGTT, plasma biochemistry and milk free fatty acids analyses were analyzed by independent *T*-test in dams. Offspring weight, base blood glucose, serum free fatty acids and plasma biochemistry were analyzed using two-way ANOVA with diet and sex as factors and Bonferroni *post-hoc* test was performed where indicated for multiple comparisons testing between groups. Differences between groups were considered significant at *P* < 0.05. All data are presented as mean ± SEM.

## Results

### Pre-pregnancy and Pregnancy Water and Caloric Intake

To identify specific differences in caloric intake between groups, 60 days prior to pregnancy and during pregnancy, water, food and caloric intake was measured daily. Total caloric intake in FD dams was significantly increased, an effect of diet, 60 days prior to pregnancy and the 69 days during pregnancy (CD, 57.94 ±2.58 vs. FD, 85.58 ± 1.98 Kcals; *P* < 0.0001) when compared to CD dams. Caloric intake from pellet food in CD and FD dams were not different. Ten percentage fructose water intake during the 60 day pre-pregnancy period and 69 days of pregnancy, on average, contributed an extra 34.0 ± 4.2 Kcal/day in fructose-fed dams. CD dams consumed, on average, 172.2 Kcal/day throughout pre-gestation and pregnancy, whereas FD dams consumed, on average, 206.2 Kcal/day with 10% fructose (w/v) contributing an extra 16.5% of total daily caloric intake in the fructose group (Table 1).

### Effects of Fructose During Pre-pregnancy and Pregnancy Weight Gain

**Figure 1:**
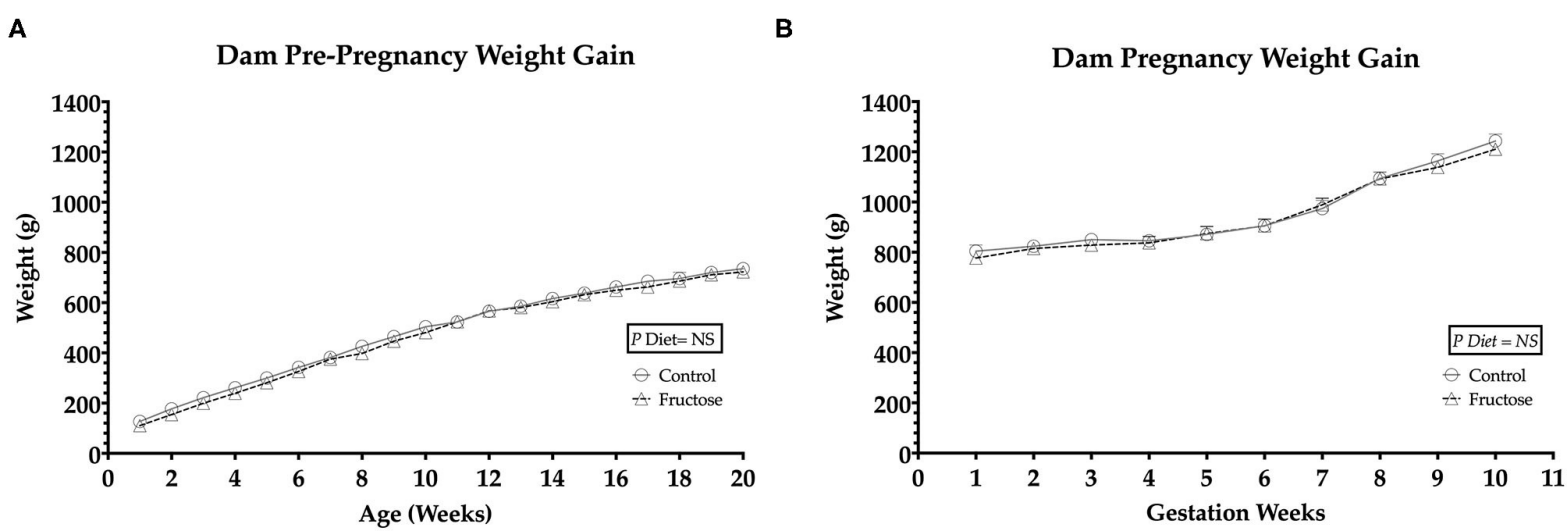
Pre-pregnancy and pregnancy weight gain during fructose-feeding **(A)** dam pre-pregnancy and **(B)** pregnancy weight gain. FD dams were fructose-fed for 60 d prior to mating and throughout pregnancy. CD (*n* = 10); FD (*n* = 9). Graph **(A)** represents average pre-pregnancy weight gain (12–20 wk), graph **(B)** represents average pregnancy weight gain (1–11 wk). All data were analyzed as a 2x2 factorial design with diet^*^time^*^interaction included (generalized linear model analysis) using IBM SPSS statistics 25 (IBM, USA). Data shown as standard error of the mean (SEM).

To observe any differences in weight gain during pre-pregnancy and pregnancy animals were weighed twice weekly. No significant differences in body weight were observed between FD and CD dams prior to (Figure 1A) or during pregnancy (Figure 1B).

### Effects of Fructose on Maternal Plasma Glucose and Insulin

**Figure 2:**
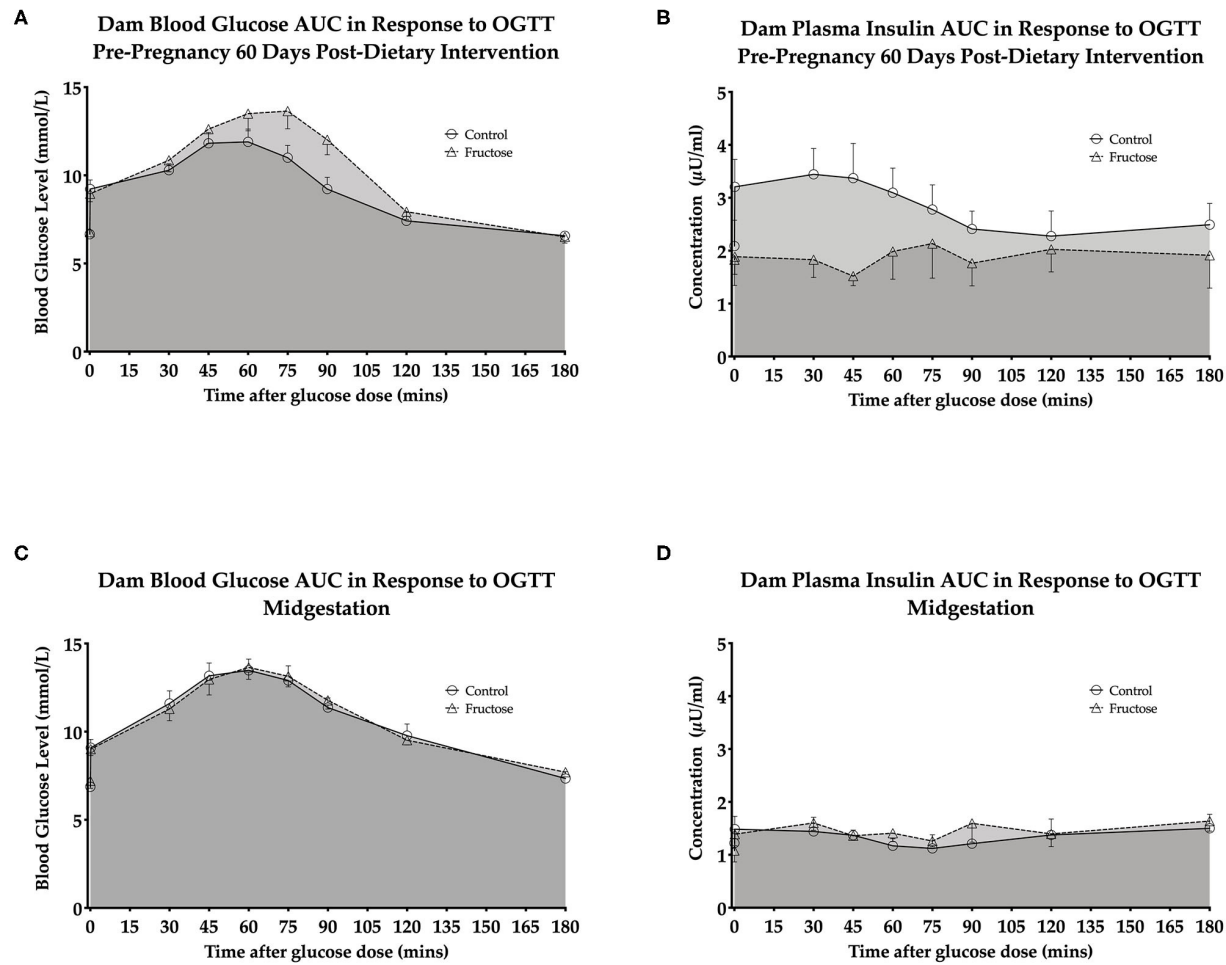
Dam pre-pregnancy and midgestation OGTT glucose AUC and insulin AUC **(A)** dam blood glucose AUC in response to OGTT pre-pregnancy 60 days post-dietary intervention. **(B)** Dam plasma insulin AUC in response to OGTT pre-pregnancy 60 days post-dietary intervention. **(C)** Dam blood glucose AUC in response to OGTT midgestation. **(D)** Dam plasma insulin AUC in response to OGTT midgestation.CD (*n* = 10); FD (*n* = 9). All data were analyzed as a 2x2 factorial design with diet*time*interaction included (generalized linear model analysis) using IBM SPSS statistics 25 (IBM, USA). Data shown as standard error of the mean (SEM).

OGTT's were performed to examine any potential effect of fructose on maternal glucose and insulin response following 60 days of consumption prior to pregnancy and during pregnancy at 35 days. A whole body insulin sensitivity index, Matsuda-DeFronzo Insulin Sensitivity Index (M-ISI), was also calculated. There was non-significant increase (*P* = 0.06)following 60 day pre-pregnancy fructose feeding in OGTT blood glucose and insulin AUC (Figures 2A,B and Supplementary Table 1) At midgestation (d35) there was no significant effect between CD and FD dams in OGTT blood glucose and insulin AUC (Figures 2C,D and Supplementary Table 1).

A non-significant increase was observed 60 days post-dietary intervention between CD and FD dams M-ISI (CD, 2.64 ± 0.27 vs. FD, 4.94 ± 1.04; *P* = 0.06) (Supplementary Table 1).

### Pre-pregnancy and Pregnancy Plasma Biochemistry

To examine the effects of fructose following 60 days of consumption prior to pregnancy and consumption during pregnancy at 35 days, plasma biochemistry was analyzed. Fructose was observed to have significant effects on dam plasma biochemistry prior to and during pregnancy. High density lipoprotein (HDL) concentrations were significantly increased, an effect of diet (CD, 0.03 ± 0.01 vs. FD, 0.15 ± 0.01 mmol/L; *P* < 0.0001), during pre-pregnancy following 60 days fructose feeding in FD dams compared to CD dams. No significant differences between FD and CD dams were observed during pregnancy. Triglycerides (TAG) were significantly increased in FD dams following 60 days of fructose feeding (CD, 0.52 ± 0.03 vs. FD, 0.69 ±0.05 mmol/L; *P* = 0.03), compared to CD dams, but this was no longer significantly different between FD and CD dams during pregnancy. No significant differences in Uric acid (UA) were observed between FD and CD dams during the 60 days of fructose feeding prior to pregnancy, whereas during midgestation there was a significant increase in uric acid of FD dams when compared to CD dams(CD, 18.66 ± 2.76 vs. FD, 21.00 ±2.76 μmol/L; *P* = 0.007) (Table 2).

**Table 2:** Dam plasma biochemistry at pre-pregnancy 60 days post-dietary intervention and midgestation.

**Dam plasma biochemistry**	**Dietary group**	**60 days Post-dietary intervention**	**Midgestation**
CHOL (mmol/L)	Control	0.64 ± 0.10	0.48 ± 0.09
	Fructose	0.64 ± 0.05	0.37 ± 0.03
LDL (mmol/L)	Control	0.71 ± 0.08	0.19 ± 0.01
	Fructose	0.72 ± 0.05	0.22 ± 0.02
HDL (mmol/L)	Control	0.03 ± 0.01	0.02 ± 0.00
	Fructose	0.15 ± 0.01^**^	0.03 ± 0.00
TAG (mmol/L)	Control	0.52 ± 0.03	1.53 ± 0.35
	Fructose	0.69 ± 0.05^*^	1.43 ± 0.29
UA (μmol/L	Control	18.66 ± 2.76	21.55 ± 1.36
	Fructose	21.00 ± 2.67	28.75 ± 1.88^**^

*CD (n = 10); FD (n = 9). All data was analyzed using an independent T-test using IBM SPSS statistics 25. Data presented as group mean ± SEM*.

* denotes significance of p < 0.05;

** *denotes significance of p < 0.001*.

### Pregnancy Outcomes

To measure the effect of fructose consumption on general pregnancy and developmental outcomes littersize, stillbirths and birth weight were measured at day of delivery. There was no significant difference between CD and FD dams in litter size, rate of stillbirth/death at delivery or offspring birth weight (Table 3).

**Table 3:** Dam pregnancy outcomes.

**Dam pregnancy outcomes**	**Day 0**	
	**Control**	**Fructose**
Litter size	3.90 ± 0.23	4.11 ± 0.20
Stillbirth/Death at delivery	0	0.44 ± 0.29
Male birth weight	90.08 ± 1.23	91.38 ± 1.23
Female weight	90.56 ± 1.23	88.71 ± 1.23
Litter birth weight	90.34 ± 1.23	90.15 ± 1.23

*Control (n = 10) and fructose (n = 9). Litter size and stillbirth/death at delivery data was analyzed using an independent T-test while birth weight was analyzed using a 2x2 factorial design with diet, sex and interaction include (general analysis of variance) using IBM SPSS statistics 25. Data presented as group mean ± SEM*.

### Maternal Milk Free Fatty Acid Composition

To examine the effects of fructose consumption on milk lipid composition a dried milk spot and lipidomic analysis were performed at day of delivery. Maternal fructose intake was observed to have significant effects on dams' milk lipid composition. From the 33 fatty acids analyzed, significant increases in FD dam milk were observed in myristic acid (CD, 1.00 ± 0.05 vs. FD, 1.16 ± 0.05 mg/100g; *P* = 0.04), total trans fatty acids (CD, 0.33 ± 0.03 vs. FD, 0.46 ± 0.036 mg/100 g; *P* = 0.02), vaccenic acid (CD, 0.15 ± 0.01 vs. FD, 0.22 ± 0.00 mg/100 g; *P* = <0.001), linoelaidic Acid (CD, 0.04 ± 0.00 vs. FD, 0.12 ± 0.02 mg/100 g; *P* = 0.002), cis-vaccenic (CD, 1.14 ± 0.06 vs. FD, 1.31 ±0.04 mg/100 g; *P* = 0.04), total omega-7 (CD, 2.39 ± 0.12 vs. FD, 2.71 ±0.08 mg/100 g; *P* = 0.05) and gamma-linolenic acid (CD, 0.06 ± 0.00 vs. FD, 0.08 ±0.00 mg/100 g; *P* = 0.05). There were no differences between CD and FD in any of the other 26 FFA concentrations measured in maternal milk (Table 4).

**Table 4:** Free fatty acids in dams' milk at day of delivery.

**Dam milk** **Free fatty acid (mg/100 g)**	**Day 0**	
	**Control**	**Fructose**
Myristic acid (14:0)	1.00 ± 0.05	1.16 ± 0.05^*^
Total trans fatty acids	0.33 ± 0.03	0.46 ± 0.03^*^
Vaccenic acid (t18:1n-7)	0.15 ± 0.01	0.22 ± 0.00^*^
Linoelaidic acid (t18:2)	0.04 ± 0.00	0.12 ± 0.02^*^
Cis-Vaccenic acid (18:1n-7)	1.14 ± 0.06	1.31 ± 0.04^*^
Total omega-7	2.39 ± 0.12	2.71 ± 0.08^*^
Gamma-Linolenic acid (18:3n-6)	0.06 ± 0.00	0.08 ± 0.00^*^
Total saturates	30.23 ± 1.31	31.64 ± 1.09
Lauric acid (12:0)	0.03 ± 0.00	0.03 ± 0.00
Pentadecanoic acid (15:0)	0.46 ± 0.02	0.52 ± 0.02
Palmitic acid (16:0)	21.35 ± 0.96	23.44 ± 1.06
Margaric acid (17:0)	0.86 ± 0.02	0.91 ± 0.03
Lignoceric acid (24:0)	0.02 ± 0.00	0.02 ± 0.00
Elaidic acid (t18:1n-9)	0.08 ± 0.00	0.09 ± 0.00
Total monos	34.49 ± 0.43	35.49 ± 0.49
Palmitoleic acid (16:1n-7)	1.24 ± 0.08	1.39 ± 0.05
Oleic acid (18:1n-9)	32.11 ± 0.55	32.28 ± 0.42
Total Omega-9	32.62 ± 0.55	32.77 ± 0.44
Total Omega-3	5.08 ± 0.34	5.34 ± 0.29
Arachidonic acid (20:4n-6)	0.22 ± 0.02	0.23 ± 0.03
Capric acid (10:0)	0.05 ± 0.00	0.04 ± 0.00
Stearic acid (18:0)	5.49 ± 0.32	5.48 ± 0.34
Arachidic acid (20:0)	0.12 ± 0.00	0.11 ± 0.00
Palmitelaidic acid (t16:1)	0.06 ± 0.02	0.04 ± 0.00
Gondoic acid (20:1n-9)	0.48 ± 0.02	0.48 ± 0.03
Alpha-Linolenic acid (18:3n-3)	5.35 ± 0.46	5.18 ± 0.30
Docosapentaenoic acid (22:5n-3)	0.10 ± 0.00	0.10 ± 0.01
Docosahexaenoic acid (22:6n-3)	0.04 ± 0.00	0.04 ± 0.00
Total Omega-6	27.61 ± 1.51	27.06 ± 0.85
Linoleic acid (18:2n-6)	26.42 ± 1.41	25.86 ± 0.77
Eicosadienoic acid (20:2n-6)	0.67 ± 0.05	0.64 ± 0.05
Dihomo-y-linolenic acid (20:3n-6)	0.17 ± 0.01	0.12 ± 0.02
Adrenic acid (22:4n-6)	0.10 ± 0.01	0.09 ± 0.015

*Control (n = 10) and fructose (n = 9). All data was analyzed using an independent T-test using IBM SPSS statistics 25. Data presented as group mean ± SEM*.

* *denotes significance of p < 0.05*.

### Postnatal Offspring Weight Gain

**Figure 3:**
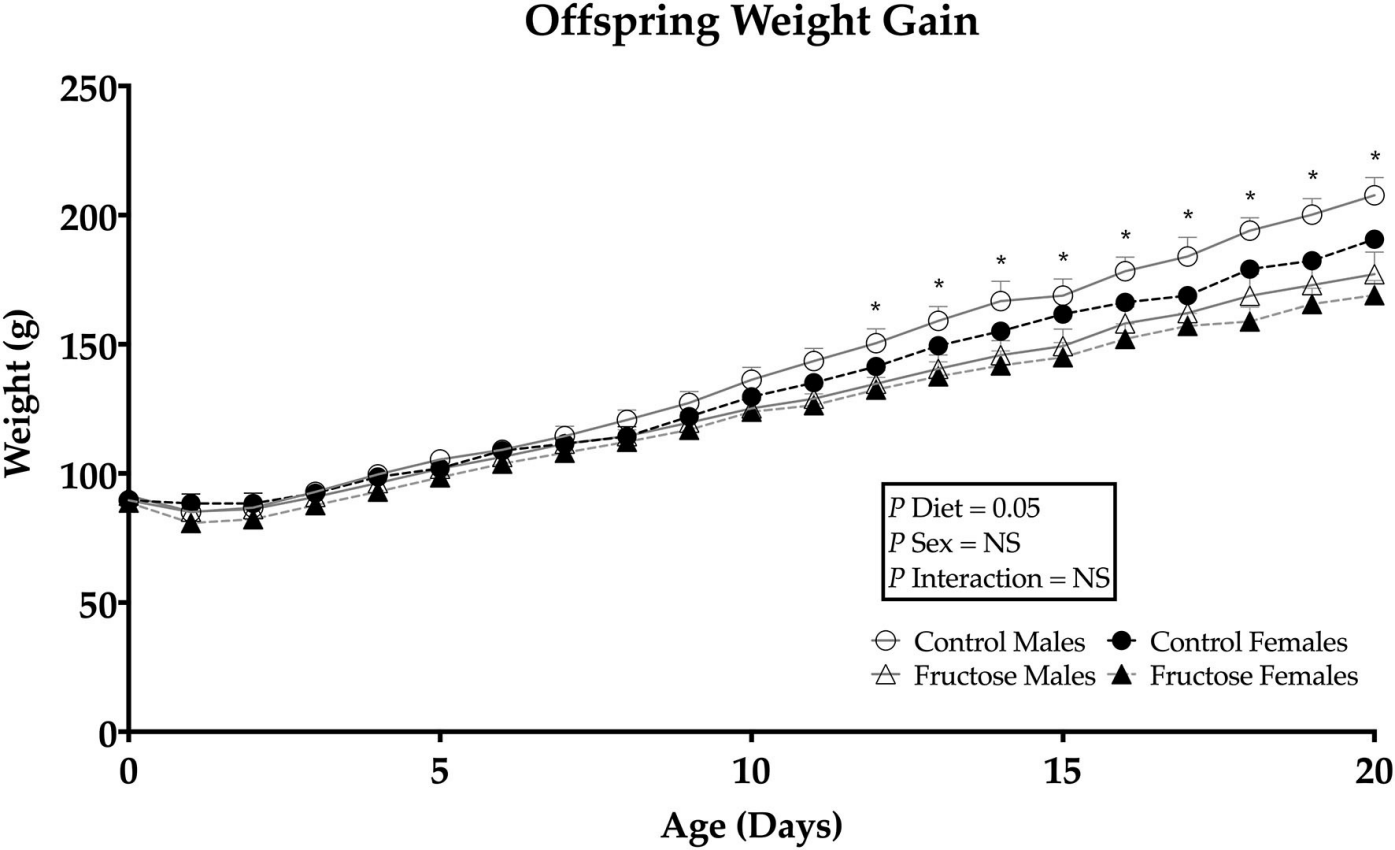
Weanling offsprings' weight gain from days 0 to 21. Control males (*n* = 7); fructose males (*n* = 7); control females (*n* = 7) and fructose females (*n* = 7). Significant effects were shown in diet of fructose offspring weight gain. All data was analyzed using a 2x2 factorial design with repeated measures, diet andsex as factors (general analysis of variance) using IBM SPSS statistics 25. Data presented as group mean ± SEM. *denotes significance of *p* < 0.05.

Offspring weights were taken daily from days 1 to 21 of age to determine any effect of dams maternal fructose intake on growth trajectory. Maternal fructose intake was observed to have an overall significant effect of diet (*P* = 0.05), on fructose offspring weight gain in both males and females. Following repeated measures analysis, FD offspring gained less weight than CD offspring from days 12 to 21of age (Figure 3).

### Offspring Effects of Maternal Fructose on Plasma Biochemistry

To examine the effects of maternal fructose on offspring plasma biochemistry markers of fructose and lipid metabolism were analyzed at day 21 of age. Fructose offspring had significantly increased plasma TAG concentrations at d21 compared to CD offspring(*P* = 0.01) (CDM, 0.51 ± 0.01 vs. FDM, 0.58 ± 0.01 vs. CDF, 0.56 ± 0.01 vs. FDF, 0.68 ± 0.01 mmol/L; *P* = 0.01). A sex effect (*P* = 0.05) was also observed, with CD and FD female offspring having increased TAG when compared to CD and FD male offspring. Similarly, plasma UA concentrations were significantly increased in FD offspring when compared to their CD counterparts (CDM, 42.33 ± 3.90 vs. FDM, 69.40 ± 3.90 vs. CDF, 46.50 ± 3.90 vs. FDF, 62.75 ± 3.90 μmol/L; *P* = 0.004). A significant effect of sex was observed in female offspring with an increase in CHOL (CDM, 0.87 ± 0.07 vs. FDM, 1.05 ± 0.07 vs. CDF, 1.50 ± 0.07 vs. FDF, 1.35 ± 0.07 mmol/L; *P* = 0.001), LDL (CDM, 0.79 ± 0.06 vs. FDM, 1.04 ± 0.06 vs. CDF, 1.25 ± 0.06 vs. FDF, 1.22 ± 0.06 mmol/L; *P* = 0.005) and HDL (CDM, 0.08 ± 0.00 vs. FDM, 0.10 ± 0.00 vs. CDF, 0.12 ± 0.00 vs. FDF, 0.13 ± 0.00 mmol/L; *P* = 0.001) (Table 5).

**Table 5:** Weanling offspring plasma biochemistry.

**Weanling offspring plasma biochemistry**	**Sex**	**Day 21**	
		**Control**	**Fructose**
CHOL (mmol/L)	Male	0.87 ± 0.07	1.05 ± 0.07
	Female	1.50 ± 0.07^*^	1.35 ± 0.07^*^
LDL (mmol/L)	Male	0.79 ± 0.06	1.04 ± 0.06
	Female	1.25 ± 0.06^*^	1.22 ± 0.06^*^
HDL (mmol/L)	Male	0.08 ± 0.00	0.10 ± 0.00
	Female	0.12 ± 0.00^**^	0.13 ± 0.00^**^
TAG (mmol/L)	Male	0.51 ± 0.01	0.58 ± 0.01^*^
	Female	0.56 ± 0.01^*^	0.68 ± 0.01^**^
UA (μmol/L	Male	42.33 ± 3.90	69.40 ± 3.90^*^
	Female	46.50 ± 3.90	62.75 ± 3.90^*^

*Control males (n = 7); fructose males (n = 7); control females (n = 7) and fructose females (n = 7). All data was analyzed using a 2x2 factorial design with diet and sex (general analysis of variance) using IBM SPSS statistics 25. Data presented as group mean ± SEM*.

* denotes significance of p < 0.05;

** *denotes significance of p < 0.001*.

### Weanling Serum Free Fatty Acid Composition

**Figure 4:**
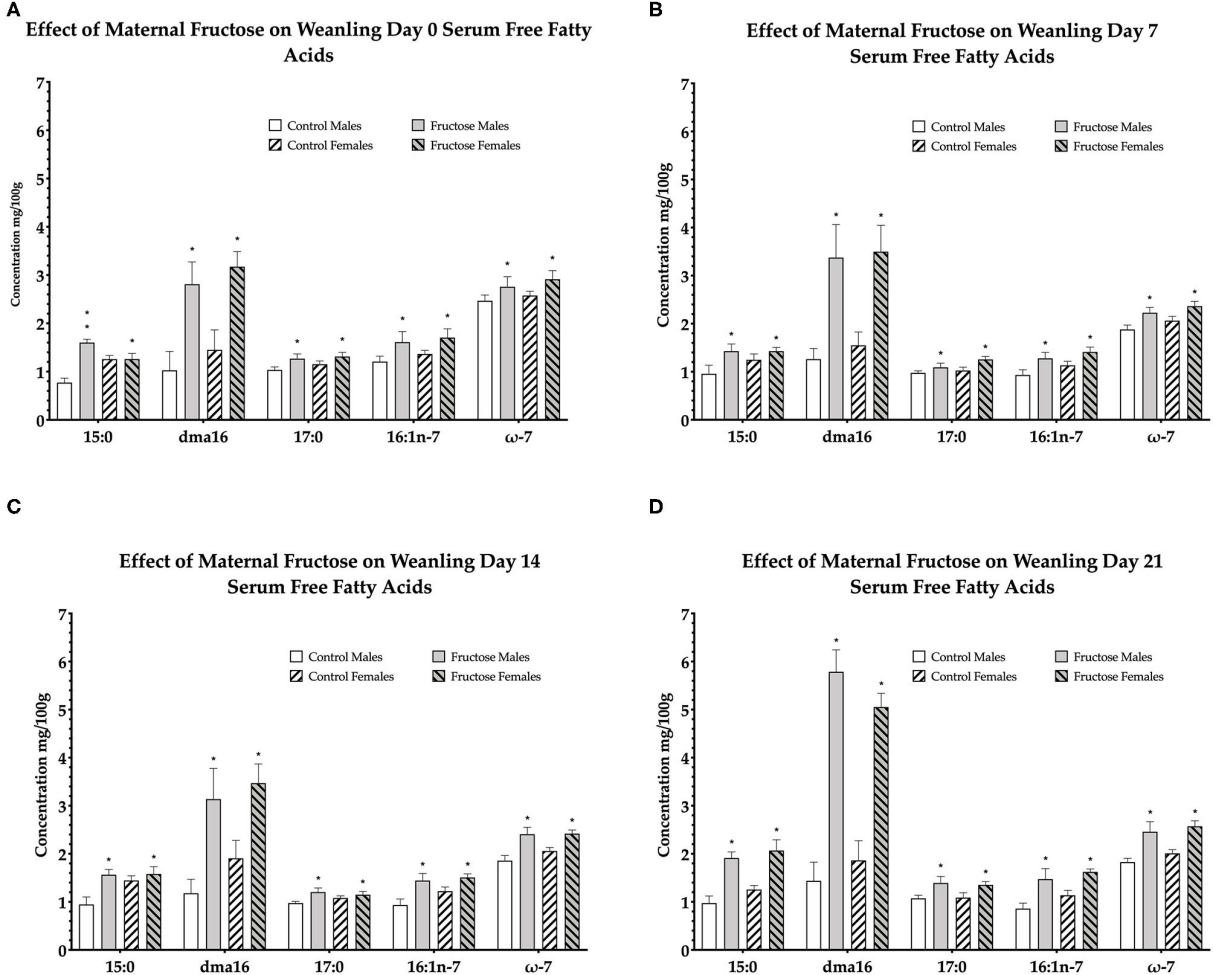
Free fatty acids in weanling offspring serum. Control males (*n* = 7); fructose males (*n* = 7); control females (*n* = 7) and fructose females (*n* = 7). **(A)** Represents offspring day 0 serum free fatty acids. **(B)** Represents offspring day 7 serum free fatty acids. **(C)** Represents offspring day 14 serum free fatty acids. **(D)** Represents offspring day 21 serum free fatty acids. All data was analyzed using a 2x2 factorial design with diet and sex as factors (general analysis of variance) using IBM SPSS statistics 25. Data presented as group mean ± SEM. *denotes significance of *p* < 0.05.

To examine the effects of maternal fructose consumption on offspring plasma lipid composition a dried blood spot and lipidomic analysis were performed at days 0, 7, 14, and 21 in offspring. Maternal fructose intake was observed to have significant effects on offspring free fatty acid composition, such that, out of the 33 serum FFAs analyzed, a significant diet effect was observed in six specific fatty acids in males and females of FD dams consistently across d0, d7, d14, and d21. Fructose males and females had significantly increased pentadecanoic acid (15:0) compared to control males and females. In fructose offspring there was an effect of diet (*P* < 0.0001) at d0, in addition, there was an interaction effect of diet and sex (*P* < 0.0001) in fructose males compared to any other group or sex. An effect of diet continued in fructose offspring at d7 (*P* = 0.02), d14 (*P* = 0.01), and d21 (*P* < 0.0001). A significant effect of diet was observed in fructose offspring dma16:0 at d0 (*P* < 0.0001), d7 (*P* < 0.0001), d14 (*P* = 0.001), and d21 (*P* < 0.0001). Margaric acid (17:0) was significantly increased in fructose offspring, an effect of diet, at d0 (*P* = 0.02), d7 (*P* = 0.02), and d14 (*P* = 0.02) and d21 (*P* = 0.005). Palmitoleic acid (16:1n-7) in fructose male and female offspring were also observed to be significantly increased, an effect of diet, compared to control at d0 (*P* = 0.02), day 7 (*P* = 0.007), d14 (*P* = 0.002), and d21 (*P* < 0.0001). A significant increase in total omega-7 (ω-7) was observed, an effect of diet, at d0 (*P* = 0.05), d7 (*P* = 0.003), d14 (*P* < 0.0001), and d21 (*P* < 0.0001) in fructose male and female offspring compared to control. In addition, a significant diet effect was observed in both FD male and female total saturates at d0 (*P* = 0.002) as well as a sex effect (*P* = 0.02) which was increased in FD females. An effect of diet continued in fructose offspring at d7 (*P* = 0.02), d14 (*P* = 0.05), and d21 (*P* < 0.0001) (d0, Figure 4A; d7, Figure 4B; d14, Figure 4C; and d21, Figure 4D).

From the remaining 27 free fatty acids analyzed, total omega-6 free fatty acids were shown to be significantly increased, an effect of diet, in control male and female offspring consistently across d0 (*P* = 0.02), d7 (*P* = 0.01), d14 (*P* = 0.05), and d21(*P* < 0.0001). The remaining free fatty acids were either non-significant or not consistently significant across all time-points.

### Effects of Fructose on Offspring Plasma Glucose

To assess the effects of maternal fructose consumption on baseline blood glucose levels analysis were performed at days 0, 7, 14, and 21 in offspring. Maternal fructose intake had a significant effect on blood glucose concentrations on d7, with an increase in FD male and female offspring (CDM, 7.40 ± 0.21 vs. FDM, 8.40 ± 0.21 vs. CDF, 7.20 ± 0.21 vs. FDF, 8.08 ± 0.21 mmol/L; *P* = 0.03) compared to their CD counterparts. However, no significant differences between CD and FD offspring were observed on d0, d14, and d21 (Table 6).

**Table 6:** Plasma biochemistry of base blood glucose in weanling offspring.

**Weanling offspring plasma biochemistry**	**Sex**	**Day 0**		**Day 7**		**Day 14**		**Day 21**	
		**Control**	**Fructose**	**Control**	**Fructose**	**Control**	**Fructose**	**Control**	**Fructose**
Base Blood Glucose (mmol/L)	Male	6.73 ± 0.16	7.02 ± 0.16	7.40 ± 0.21	8.40 ± 0.21^*^	7.50 ± 0.21	7.48 ± 0.21	7.97 ± 0.12	7.71 ± 0.12
	Female	6.00 ± 0.16	6.65 ± 0.16	7.20 ± 0.21	8.08 ± 0.21^*^	6.83 ± 0.21	7.00 ± 0.21	7.68 ± 0.12	7.22 ± 0.12

*Control males (n = 7); fructose males (n = 7); control females (n = 7) and fructose females (n = 7). All data was analyzed using a 2x2 factorial design with diet, sex and interaction include (general analysis of variance) using IBM SPSS statistics 25. Data presented as group mean ± SEM*.

* *denotes significance of p < 0.05*.

## Discussion

We have investigated the effects of moderate fructose intake on the metabolic status of dams prior to and during pregnancy and subsequent offspring metabolic status up to weaning. We hypothesized that changes in maternal nutritional status following fructose intake would alter dams metabolic profiles and milk lipid composition and that offspring metabolic profiles also display signs of hyperlipidemia. We show that an intake of fructose which closely resembles average human consumption, contributing 16.5% of total caloric intake during pregnancy, has a significant impact upon a pregnant dams' metabolic status and negatively impacts milk lipid composition. We also provide the first evidence that offspring born from fructose-fed dams displaying a very specific pattern of increased FFAs at multiple time points (d0, d7, d14, & d21). Similar to previous studies, increases in triglycerides and placental uric acid were also observed in male and female offspring (33). These potentially deleterious changes in offspring metabolic status and weight gain may likely be an effect of *in utero* exposure to maternal fructose consumption.

With similar placental characteristics to humans, relatively long gestation and comparable developmental maturation of organ systems before birth (34), the guinea pig is a relevant translational model to study the effects of maternal fructose intake on offspring development. In the current study and previously shown in the rat model, we show that water intake increased in fructose mothers, due to the addition of 10% fructose (w/v), making the water sweeter and more palatable (11, 35). All mothers consumed, on average, 172 Kcal/day from pelleted food while fructose-fed mothers ingested an extra 34 Kcal/day (16.5% total daily Kcal) from 10% fructose (w/v). Recent data has shown that human average caloric intake from fructose can be between ~10 and 20% (15). In a human context, consuming a basal diet of 2,000 kcal/day with the addition of one 16 oz sugar-sweetened beverage would equate to an increase of up to 10% of total kcal/day intake. It has been recently reported that consumption of sugar by pregnant women equates to around 14% of their average daily caloric intake (15). Animal studies of fructose intake have ranged from the supraphysiological (30–70%); (15, 36, 37) to the more comparable ranges of 10–20% fructose (w/v) (15, 18, 20, 38, 39).

Animal and human studies have shown that continued excessive dietary fructose intake increases activity of lipogenic liver enzymes, lipid synthesis and circulating LDL, VLDL, HDL and triglyceride concentrations in blood as fructose favors lipogenesis (8, 9, 40, 41). We show that prior to pregnancy, fructose intake increased levels of triglycerides and HDL without an increase in LDL but these differences were abolished during pregnancy. Similar to the current study, others have also shown no change in plasma triglycerides, HDL and LDL during pregnancy following fructose-feeding (42). The relationship between dietary fructose and insulin resistance in humans is uncertain as excess dietary fructose does not invariably cause elevated insulin concentrations (43). Bezerra et al. showed increased insulin in response to fructose-feeding and Vickers et al. published reports of fructose increasing insulin in rats (20, 44). In contrast, we show non-significant increase in blood glucose AUC and reduced insulin response AUC in dams exposed to fructose enriched diets. This has been shown previously (45) and may involve reduced insulin response due to higher concentrations of FFAs, triglycerides, HDLs and LDLs (46, 47). Our study uses a more physiologically relevant fructose intake (10%) and thus, the increases in plasma lipids observed in our non-pregnant dams could have been ameliorated by blood volume expansion, increased metabolic demand that is placed upon the expectant mother, increased hepatic gluconeogenesis and FFA production, typically observed during pregnancy (48).

Of particular interest to our study, is milk FFA composition and the potential negative impact of altered milk composition on the vertical transmission of FFAs from mother to offspring. Research has shown that milk composition can be selectively affected by maternal nutritional status and even more so during lactation. Two recent studies have shown that fructose is transferred from mother to infant during breastfeeding (49, 50), and a positive association between level of fructose in breast milk and infant adiposity at 6 months of age. In the present study, significantly increased FFAs in maternal milk included myristic acid (14:0), total trans fatty acids, vaccenic acid (t18:1n-7), linoelaidic acid (t18:2) cis-vaccenic acid (18:1n-7) total omega-7 and gamma-linolenic acid (18:3n-6), the majority of which are trans-fats. Studies have demonstrated that trans fatty acid content in lactating mothers is both directly associated with diet and independent of diet through maternal adipose tissue (51, 52).

Despite dams not having access to fructose during lactation in the current study, we also show a reduced pup weight at d12–21. Zou et al. reported a reduction in pup weight over time from mothers that were fed fructose (63%) during pregnancy and lactation (53). Similarly, Rawana et al. reported reduced body weight and total litter weight at weaning in offspring of dams consuming 10% fructose (w/v) during pregnancy and lactation (19). In contrast, Alzamendi et al. reported that at d20 of gestation fetal:placental ratio was increased in dams fed 10% (w/v) fructose solution throughout pregnancy, suggesting that fructose offspring were heavier than the control group (54). There is evidence to suggest that fructose intake is linked with inappropriate vascularisation of the placenta and further studies have shown irregularities in the zonal regions of the placenta (20). This may suggest a potential mechanism by which maternal fructose intake has deleterious effects on vascularisation in the placenta and nutrient transfer, contributing to reducing neonatal growth trajectory. We would suggest that following breastfeeding, specific changes in milk FFA composition, as a result of gestational fructose intake, may further contribute to the already increased circulating FFA profile observed in fructose offspring. This may indicate an additional route by which offspring are further exposed to the deleterious effects of maternal fructose-feeding despite the mothers never receiving fructose during lactation.

The deleterious effects of elevated plasma uric acid concentrations following fructose consumption in non-pregnant subjects has been well established (55, 56). Some research, but not all, has shown excessive maternal fructose intake to induce increased uric acid production during pregnancy by increasing adenosine triphosphate (ATP) degradation to adenosine monophosphate (AMP), a uric acid precursor (33, 56, 57). Similar effects of maternal fructose intake are evident in our pre-pregnant and pregnant dams, with a significant increase in uric acid before pregnancy and during pregnancy. Moreover, we also show significantly increased plasma uric acid and triglycerides in fructose offspring at weaning, despite these pups never consuming fructose themselves. These results are likely to be a consequence of (a) fructose dams and offspring producing high concentrations of uric acid due to the metabolic effects of higher circulating FFAs, which is causal of increased intracellular ATP resulting from excessive fructose (56, 57), and/or (b) uric acid is transferred to the fetus during pregnancy from the mother via the placenta. Uric acid has been shown to cross the placenta freely and increased exposure to uric acid *in utero* may potentially affect physiological set-points in the developing fetus. In the current study, increased uric acid observed in offspring are likely to also be a consequence of dyslipidemia and unregulated circulating FFAs and potentially, dysfunctional beta-oxidation and increased accumulation of intracellular ATP in the offspring. However, which pathway is more likely to be playing the primary role in the current study is unknown at this stage and further work to examine the molecular nature of such significant findings must be performed.

FFAs during fasting or in the absence of glucose are released from adipose tissue and are primarily metabolized by skeletal muscle and the liver (58). It is well established that chronic increased levels of circulating FFAs can increase the risk of obesity, hepatic lipid deposition, insulin resistance, type 2 diabetes and cardiovascular disease (59) and increased fatty acid synthesis has been shown to occur following fructose consumption (60–62). The effects of fructose on fatty acid synthesis is likely to be dose-dependent, due to the lipogenic effects of fructose. Long-term increases in circulating FFAs have also been shown in offspring of mothers fed diets high in sucrose and fructose (HFC-55). Toop et al. showed increased hepatic lipid composition and content in fructose offspring (63). They went on to hypothesize that increased hepatic *de novo* lipogenesis was caused, in part, by increased circulating FFAs. In the current study, increases in FFAs were observed in pentadecanoic acid (15:0), dma16:0, margaric acid (17:0) palmitoleic acid, total omega-7 and total saturates. More importantly, fructose offspring serum displayed these specific changes in serum FFAs at day 0, when samples were taken prior to their first feed and before receiving any milk from the mother. These increases in circulating FFAs were consistently significantly higher at all other time points (days 7, 14, and 21). This clearly indicates an *in utero* metabolic programming of offspring FFA metabolism. Our findings are of particular importance because of the consistency in the increase of specific FFAs observed. As prolonged FFAs may also be present as a consequence of dysfunctional adipose lipolysis and altered beta-oxidation function (59). Additionally, the specific type of FFA increases also has important metabolic implications (64). The majority of the significantly increased FFAs observed in fructose offspring and maternal milk were long-chain fatty acids which are primary components of storage triglycerides.

The majority of offspring plasma saturated fats are associated with hyperglycemia, hyperinsulinemia and insulin resistance. Chronic elevations of circulating FFAs augment fat storage, insulin resistance and development of metabolic disease (59). Our results provide evidence of maternal fructose increasing *de novo* lipogenesis, shown by increased circulating triglycerides, uric acid, LDL and HDL prior to pregnancy, changes in milk lipid composition at birth, perturbed offspring metabolic status and potential predisposition to metabolic disease in later-life. A limitation of the current study is a lack of molecular and lipogenic enzymic analysis in offspring. Few studies have investigated similar models to our own. However, a previous study by Clayton et al., showed that genes related to hepatic lipogenesis (SPBP1c) were increased, showing a phenotype with differential expression in lipogenic gene expression. It would be interesting to determine hepatic lipogenic enzyme activity and activation of molecular gene expression to investigate a “true” programming or causal effects of maternal fructose intake and preturbed offspring hepatic metabolism. Nevertheless, this study focused on the primary outcomes of maternal and offspring phenotype where we show, in principle, at multiple time points, a perinatally acquired predisposition to metabolic dysfunction in offspring. While the precise mechanism driving the offspring hyperlipidemia and deleterious FFA profile is not fully understood, the current study clearly demonstrates how maternal fructose can influence the developmental programming of offspring lipid metabolism during critical windows of plasticity prior to suckling, which may alter an individual's later-life susceptibility to metabolic disease. However, more studies are needed to clarify the potential gene and functional enzymatic pathways which are preturbed in models of maternal fructose intake.

In conclusion, we have shown for the first time, highly significant, consistent changes in young offspring FFA serum profiles, despite offspring consuming no fructose themselves. A specific pattern of increased FFAs were present prior to suckling d0 and to d21 of age, as well as increased uric acid and triglycerides in offspring of fructose-fed dams. Taken together, we provide evidence that indicates unregulated *de novo* fatty acid synthesis in offspring from mothers fed a moderate 10% (w/v) fructose water prior to and during pregnancy. We would suggest that suboptimal maternal diets, in particular, those high in fructose and refined sugars contribute to the rise in metabolic diseases observed over the last 40–50 years. Our study, along with other current research, emphasizes the importance of limiting added refined fructose such as sugar-sweetened beverage intake and striving for a more nutritionally-balanced diet in women prior to/during pregnancy and lactation.

## Data Availability Statement

The raw data supporting the conclusions of this article will be made available by the authors, without undue reservation.

## Ethics Statement

The animal study was reviewed and approved by University of Otago Animal Ethics Committee.

## Author Contributions

CG designed research and funding acquisition. ES, RD, and CG conducted research. ES wrote the first draft manuscript. RD, MB, and CG critically evaluated the paper. All authors contributed to the article and approved the submitted version.

## Conflict of Interest

The authors declare that the research was conducted in the absence of any commercial or financial relationships that could be construed as a potential conflict of interest.

## Supplementary Material

Supplementary Table 1 Effects of fructose on dam 60-days post-dietary intervention and midgestation OGTT Gluose, Insulin and Matsuda-ISI CD (*n* = 6); FD (*n* = 6). All data was analyzed using an independent *T*-test using IBM SPSS statistics 25. Data presented as group mean ± SEM.

## Abbreviations

CD: Control diet
FD: Fructose diet
OGTT: Oral Glucose Tolerance Test
CHOL: Cholesterol
LDL: low-density lipoprotein cholesterol
HDL: high-density lipoprotein cholesterol
UA: Uric Acid
TAG: triglycerides
DBS: dried blood spot
DMS: dried milk spot
NAFLD: non-alcoholic fatty liver disease
M-ISI: Matsuda-DeFronzo insulin sensitivity index.
