# Supplementary material for: Fructose Consumption During Pregnancy Influences Milk Lipid Composition and Offspring Lipid Profiles in Guinea Pigs
Source: Front Endocrinol (Lausanne). 2020 Aug 11;11:550. doi: 10.3389/fendo.2020.00550 (PMC7431635; doi:10.3389/fendo.2020.00550)
Supplement: Supplementary Table 1 — Effects of fructose on dam 60-days post-dietary intervention and midgestation OGTT Gluose, Insulin and Matsuda-ISI CD (n = 6); FD (n = 6). All data was analyzed using an independent T-test using IBM SPSS statistics 25. Data presented as group mean ± SEM. [file Table_1.pdf]

Dam OGTT Response	Dietary Group	60 Days Post-Dietary Intervention	*P*-value	Midgestation	*P*-value
**Blood Glucose (mmol/L)**					
Baseline	Control	6.66 ±0.12	0.64	6.87 ±0.19	0.45
	Fructose	6.77 ±0.20		7.21 ±0.42	
30mins	Control	10.28 ±0.36	0.46	11.60 ±0.71	0.75
	Fructose	10.85 ±0.65		11.28 ±0.66	
45mins	Control	11.82 ±0.60	0.43	13.17 ±0.72	0.85
	Fructose	12.62 ±0.81		12.96 ±0.87	
60mins	Control	11.90 ±0.72	0.20	13.48 ±0.63	0.86
	Fructose	13.50 ±0.97		13.64 ±0.67	
75mins	Control	11.00 ±0.69	0.04	12.90 ±0.83	0.83
	Fructose	13.64 ±1.00*		13.14 ±0.60	
90mins	Control	9.22 ±0.66	0.01	11.36 ±0.54	0.59
	Fructose	12.02 ±0.85*		11.77 ±0.49	
120mins	Control	7.42 ±0.46	0.44	9.77 ±0.65	0.72
	Fructose	7.93 ±0.43		9.51 ±0.27	
180mins	Control	6.57 ±0.17	0.88	7.34 ±0.39	0.49
	Fructose	6.51 ±0.35		7.70 ±0.25	
**Plasma Insulin (μU/ml)**					
Baseline	Control	2.08 ±0.48	0.71	1.22 ±0.25	0.68
	Fructose	1.82 ±0.48		1.08 ±0.21	
30min	Control	3.44 ±0.48*	0.02	1.44 ±026	0.60
	Fructose	1.82 ±0.33		1.60 ±0.13	
45min	Control	3.37 ±0.65*	0.02	1.36 ±0.09	0.95
	Fructose	1.51 ±0.18		1.36 ±0.07	
60mins	Control	3.09 ±0.46	0.14	1.17 ±0.13	0.27
	Fructose	1.98 ±0.52		1.40 ±0.15	
75mins	Control	2.77 ±0.46	0.44	1.11 ±0.25	0.62
	Fructose	2.13 ±0.65		1.26 ±0.10	
90mins	Control	2.41 ±0.33	0.26	1.21 ±0.37	0.47
	Fructose	1.76 ±0.42		1.59 ±0.35	
120mins	Control	2.27 ±0.47	0.70	1.37 ±0.30	0.95
	Fructose	2.02 ±0.42		1.39 ±0.24	
180mins	Control	2.49 ±0.40	0.45	1.50 ±0.26	0.68
	Fructose	1.91 ±0.61		1.63 ±0.18	
**Matsuda-ISI**	Control	2.64 ±0.27	0.06	4.94 ±1.04	0.12
	Fructose	4.94 ±1.04		5.99 ±0.45	
